# Phenazin-5-ium bromide

**DOI:** 10.1107/S1600536812027869

**Published:** 2012-06-23

**Authors:** Gong-Xiao Zhang, Ping Li, Jian Dong, Hong-Yu Chen

**Affiliations:** a Faculty of Chemistry and Chemical Engineering, TaiShan Medical University, Tai’an 271016, People’s Republic of China

## Abstract

In the title compound, C_12_H_9_N_2_^+^·Br^−^, the protonated tricyclic ring system is slightly twisted, with a dihedral angle of 3.9 (1)° between the two outer benzene rings. In the crystal, N—H⋯Br and C—H⋯Br hydrogen bonds link two cations and two bromide anions into centrosymmetric assemblies, which are further packed into stacks along [010] *via* π–π inter­actions between the aromatic rings [centroid–centroid distance = 3.725 (4) Å].

## Related literature

For applications of phenazines, see: Laursen & Nielsen (2004 ▶); Uchida & Kimura (1984 ▶). For related structures, see: Braga *et al.* (2010 ▶); Zhang *et al.* (2012 ▶).
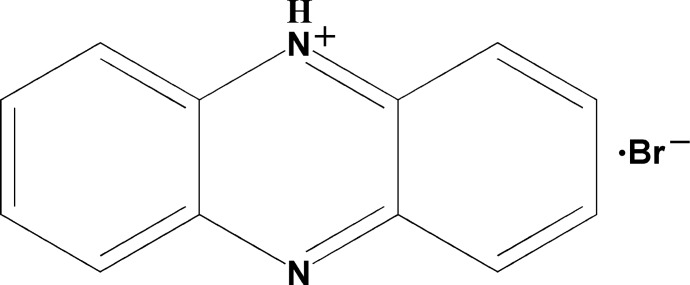


## Experimental

### 

#### Crystal data

C_12_H_9_N_2_^+^·Br^−^
*M* *_r_* = 261.12
Triclinic, 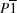

*a* = 5.639 (5) Å
*b* = 7.958 (5) Å
*c* = 12.149 (5) Å
α = 73.284 (5)°
β = 86.896 (5)°
γ = 88.360 (5)°
*V* = 521.3 (6) Å^3^
*Z* = 2
Mo *K*α radiation
μ = 3.91 mm^−1^
*T* = 293 K
0.18 × 0.16 × 0.15 mm

#### Data collection

Bruker SMART APEXII CCD area-detector diffractometer
Absorption correction: multi-scan (*SADABS*; Bruker, 2005 ▶) *T*_min_ = 0.541, *T*_max_ = 0.556
3029 measured reflections
2085 independent reflections
1840 reflections with *I* > 2σ(*I*)
*R*_int_ = 0.028

#### Refinement

*R*[*F* ^2^ > 2σ(*F* ^2^)] = 0.038
*wR*(*F* ^2^) = 0.105
*S* = 1.08
2085 reflections
137 parameters
H-atom parameters constrained
Δρ_max_ = 0.81 e Å^−3^
Δρ_min_ = −0.65 e Å^−3^

### 

Data collection: *APEX2* (Bruker, 2005 ▶); cell refinement: *SAINT* (Bruker, 2005 ▶); data reduction: *SAINT*; program(s) used to solve structure: *SIR97* (Altomare *et al.*, 1999 ▶); program(s) used to refine structure: *SHELXL97* (Sheldrick, 2008 ▶); molecular graphics: *XP* in *SHELXTL* (Sheldrick, 2008 ▶); software used to prepare material for publication: *WinGX* (Farrugia, 1999 ▶).

## Supplementary Material

Crystal structure: contains datablock(s) global, I. DOI: 10.1107/S1600536812027869/cv5312sup1.cif

Structure factors: contains datablock(s) I. DOI: 10.1107/S1600536812027869/cv5312Isup2.hkl

Supplementary material file. DOI: 10.1107/S1600536812027869/cv5312Isup3.cml

Additional supplementary materials:  crystallographic information; 3D view; checkCIF report

## Figures and Tables

**Table 1:** Hydrogen-bond geometry (Å, °)

*D*—H⋯*A*	*D*—H	H⋯*A*	*D*⋯*A*	*D*—H⋯*A*
N2—H2*B*⋯Br1	0.86	2.31	3.155 (4)	167
C3—H3*A*⋯Br1^i^	0.93	2.82	3.750 (5)	176

Symmetry code: (i) 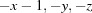.

**Fig. 1.**
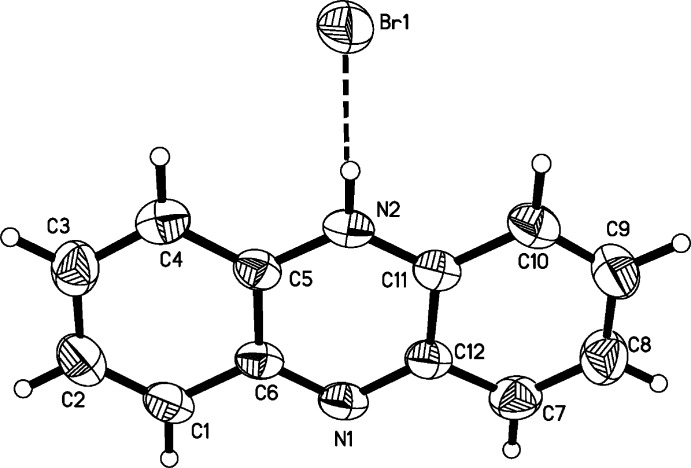
The molecular structure of (I) showing the atomic numbering and 50% probability displacement ellipsoids. Dashed line denotes hydrogen bond.

## supplementary crystallographic information

### Comment

In the past decade, much interest has been focused on the phenazine as a template in crystal engineering. The electron rich aromatic system in phenazine enables it to be a good π-donor. Accordingly, phenazine has been employed in the design of charge-transfer complexes (Laursen *et al.*, 2004; Uchida *et al.*, 1984). In a continuation of our study of the compounds with phenazinium cation (Zhang *et al.*, 2012), we present here the title compound, (I).

In (I) (Fig. 1), the bond lengths and angles are normal and correspond to those observed in the related phenazinium chloride (Braga *et al.*, 2010). The asymmetric unit of (I) contains a phenazinium cation and a bromide anion. The phenazinium cations show planar configuration with the largest deviation from the least-square-plane of 0.053 (4) Å for C7. The protonated tricycle is twisted with a dihedral angle of 3.9 (1)° between the two utmost benzene rings.

The cations are packed along the *b* axis and the tilted angle between the phenazinium plane and *b* axis of 50.40 (5)°. In the crystal, N—H···Br and C—H···Br hydrogen bonds (Table 1) link two cations and two bromide anions into centrosymmetric clusters, which are further packed into stacks along [010] *via*π–π interactions between the aromatic rings [centroid-centroid distance = 3.725 (4) Å].

### Experimental

Phenazine(10.0 g) and 2-bromopropane (4.2 mL) was placed in the teflon liner of an autoclave, which was sealed and heated to 433 K for 48 h, cooled at speed of 10 K/min, whereupon a few of black block of title crystal were obtained.

### Refinement

All H atoms were geometrically positioned (C—H = 0.93 Å, N—H = 0.86 Å), and allowed to ride on their parent atoms, with *U*_iso_(H)= 1.2*U*_eq_(C, N).

### Crystal data

**Table:**

C_12_H_9_N_2_^+^·Br^−^	*Z* = 2
*M**_r_* = 261.12	*F*(000) = 260
Triclinic, *P*1	*D*_x_ = 1.663 Mg m^−^^3^
Hall symbol: -P 1	Mo *K*α radiation, λ = 0.71069 Å
*a* = 5.639 (5) Å	Cell parameters from 1973 reflections
*b* = 7.958 (5) Å	θ = 2.7–28.3°
*c* = 12.149 (5) Å	µ = 3.91 mm^−^^1^
α = 73.284 (5)°	*T* = 293 K
β = 86.896 (5)°	Prism, black
γ = 88.360 (5)°	0.18 × 0.16 × 0.15 mm
*V* = 521.3 (6) Å^3^	

### Data collection

**Table:**

Bruker SMART APEXII CCD area-detector diffractometer	2085 independent reflections
Radiation source: fine-focus sealed tube	1840 reflections with *I* > 2σ(*I*)
Graphite monochromator	*R*_int_ = 0.028
phi and ω scans	θ_max_ = 26.4°, θ_min_ = 2.7°
Absorption correction: multi-scan (*SADABS*; Bruker, 2005)	*h* = −4→7
*T*_min_ = 0.541, *T*_max_ = 0.556	*k* = −9→9
3029 measured reflections	*l* = −15→14

### Refinement

**Table:**

Refinement on *F*^2^	Secondary atom site location: difference Fourier map
Least-squares matrix: full	Hydrogen site location: inferred from neighbouring sites
*R*[*F*^2^ > 2σ(*F*^2^)] = 0.038	H-atom parameters constrained
*wR*(*F*^2^) = 0.105	*w* = 1/[σ^2^(*F*_o_^2^) + (0.062*P*)^2^ + 0.0297*P*] where *P* = (*F*_o_^2^ + 2*F*_c_^2^)/3
*S* = 1.08	(Δ/σ)_max_ < 0.001
2085 reflections	Δρ_max_ = 0.81 e Å^−^^3^
137 parameters	Δρ_min_ = −0.65 e Å^−^^3^
0 restraints	Extinction correction: *SHELXL97* (Sheldrick, 2008), Fc^*^=kFc[1+0.001xFc^2^λ^3^/sin(2θ)]^-1/4^
Primary atom site location: structure-invariant direct methods	Extinction coefficient: 0.028 (5)

### Special details

**Table:**

Geometry. All e.s.d.'s (except the e.s.d. in the dihedral angle between two l.s. planes) are estimated using the full covariance matrix. The cell e.s.d.'s are taken into account individually in the estimation of e.s.d.'s in distances, angles and torsion angles; correlations between e.s.d.'s in cell parameters are only used when they are defined by crystal symmetry. An approximate (isotropic) treatment of cell e.s.d.'s is used for estimating e.s.d.'s involving l.s. planes.
Refinement. Refinement of *F*^2^ against ALL reflections. The weighted *R*-factor *wR* and goodness of fit *S* are based on *F*^2^, conventional *R*-factors *R* are based on *F*, with *F* set to zero for negative *F*^2^. The threshold expression of *F*^2^ > σ(*F*^2^) is used only for calculating *R*-factors(gt) *etc*. and is not relevant to the choice of reflections for refinement. *R*-factors based on *F*^2^ are statistically about twice as large as those based on *F*, and *R*- factors based on ALL data will be even larger.

### Fractional atomic coordinates and isotropic or equivalent isotropic displacement parameters (Å^2^)

**Table:**

	*x*	*y*	*z*	*U*_iso_*/*U*_eq_	
Br1	−0.54425 (5)	−0.08849 (4)	0.30612 (2)	0.04706 (19)	
C1	0.2320 (6)	0.3452 (4)	−0.0468 (3)	0.0430 (7)	
H1A	0.3740	0.4032	−0.0732	0.052*	
C2	0.1020 (6)	0.2915 (5)	−0.1191 (3)	0.0494 (8)	
H2A	0.1559	0.3123	−0.1955	0.059*	
C3	−0.1152 (6)	0.2040 (5)	−0.0819 (3)	0.0492 (8)	
H3A	−0.2033	0.1706	−0.1345	0.059*	
C4	−0.1977 (6)	0.1679 (4)	0.0300 (3)	0.0435 (7)	
H4A	−0.3401	0.1095	0.0543	0.052*	
C5	−0.0634 (5)	0.2204 (4)	0.1074 (2)	0.0354 (6)	
C6	0.1524 (5)	0.3133 (4)	0.0701 (2)	0.0353 (6)	
C7	0.3209 (6)	0.4035 (5)	0.3284 (3)	0.0481 (8)	
H7A	0.4564	0.4703	0.3029	0.058*	
C8	0.2429 (7)	0.3683 (5)	0.4392 (3)	0.0544 (9)	
H8A	0.3257	0.4105	0.4897	0.065*	
C9	0.0368 (7)	0.2680 (5)	0.4799 (3)	0.0525 (9)	
H9A	−0.0118	0.2442	0.5571	0.063*	
C10	−0.0911 (5)	0.2059 (4)	0.4102 (3)	0.0433 (7)	
H10A	−0.2270	0.1408	0.4382	0.052*	
C11	−0.0137 (5)	0.2419 (4)	0.2938 (2)	0.0351 (6)	
C12	0.1979 (5)	0.3395 (4)	0.2505 (2)	0.0363 (6)	
N1	0.2781 (4)	0.3709 (3)	0.1416 (2)	0.0384 (6)	
N2	−0.1337 (4)	0.1862 (3)	0.2191 (2)	0.0364 (5)	
H2B	−0.2603	0.1264	0.2435	0.044*	

### Atomic displacement parameters (Å^2^)

**Table:**

	*U*^11^	*U*^22^	*U*^33^	*U*^12^	*U*^13^	*U*^23^
Br1	0.0445 (3)	0.0526 (3)	0.0440 (2)	−0.01652 (15)	0.00696 (14)	−0.01376 (15)
C1	0.0355 (16)	0.0472 (18)	0.0418 (16)	0.0064 (13)	0.0064 (13)	−0.0076 (13)
C2	0.055 (2)	0.051 (2)	0.0394 (17)	0.0125 (16)	0.0031 (15)	−0.0116 (14)
C3	0.055 (2)	0.050 (2)	0.0484 (18)	0.0097 (16)	−0.0095 (15)	−0.0221 (15)
C4	0.0348 (16)	0.0428 (17)	0.0528 (18)	0.0010 (13)	−0.0036 (13)	−0.0136 (14)
C5	0.0311 (14)	0.0353 (15)	0.0385 (15)	0.0048 (12)	0.0017 (11)	−0.0096 (11)
C6	0.0279 (14)	0.0345 (15)	0.0401 (15)	0.0040 (12)	0.0015 (11)	−0.0062 (12)
C7	0.0362 (18)	0.0523 (19)	0.0532 (19)	−0.0104 (15)	0.0010 (14)	−0.0106 (15)
C8	0.058 (2)	0.062 (2)	0.0476 (19)	−0.0110 (18)	−0.0046 (15)	−0.0205 (16)
C9	0.057 (2)	0.060 (2)	0.0393 (17)	−0.0022 (17)	0.0052 (15)	−0.0127 (15)
C10	0.0381 (17)	0.0447 (18)	0.0437 (17)	−0.0050 (14)	0.0076 (13)	−0.0087 (13)
C11	0.0306 (14)	0.0340 (15)	0.0383 (15)	0.0006 (11)	0.0024 (11)	−0.0072 (11)
C12	0.0295 (15)	0.0368 (15)	0.0389 (15)	0.0003 (12)	0.0014 (11)	−0.0056 (12)
N1	0.0283 (12)	0.0398 (14)	0.0426 (13)	−0.0009 (10)	0.0043 (10)	−0.0056 (10)
N2	0.0263 (12)	0.0381 (13)	0.0430 (13)	−0.0042 (10)	0.0053 (10)	−0.0096 (10)

### Geometric parameters (Å, º)

**Table:**

C1—C2	1.339 (5)	C7—C12	1.417 (5)
C1—C6	1.418 (4)	C7—H7A	0.9300
C1—H1A	0.9300	C8—C9	1.413 (5)
C2—C3	1.413 (5)	C8—H8A	0.9300
C2—H2A	0.9300	C9—C10	1.344 (5)
C3—C4	1.364 (4)	C9—H9A	0.9300
C3—H3A	0.9300	C10—C11	1.407 (4)
C4—C5	1.397 (5)	C10—H10A	0.9300
C4—H4A	0.9300	C11—N2	1.339 (4)
C5—N2	1.345 (4)	C11—C12	1.433 (4)
C5—C6	1.426 (4)	C12—N1	1.329 (4)
C6—N1	1.336 (4)	N2—H2B	0.8600
C7—C8	1.345 (5)		
			
C2—C1—C6	119.8 (3)	C12—C7—H7A	119.8
C2—C1—H1A	120.1	C7—C8—C9	120.8 (3)
C6—C1—H1A	120.1	C7—C8—H8A	119.6
C1—C2—C3	121.5 (3)	C9—C8—H8A	119.6
C1—C2—H2A	119.2	C10—C9—C8	122.0 (3)
C3—C2—H2A	119.2	C10—C9—H9A	119.0
C4—C3—C2	121.0 (3)	C8—C9—H9A	119.0
C4—C3—H3A	119.5	C9—C10—C11	118.2 (3)
C2—C3—H3A	119.5	C9—C10—H10A	120.9
C3—C4—C5	118.6 (3)	C11—C10—H10A	120.9
C3—C4—H4A	120.7	N2—C11—C10	121.6 (3)
C5—C4—H4A	120.7	N2—C11—C12	117.3 (3)
N2—C5—C4	121.3 (3)	C10—C11—C12	121.2 (3)
N2—C5—C6	117.8 (3)	N1—C12—C7	120.4 (3)
C4—C5—C6	120.9 (3)	N1—C12—C11	122.3 (3)
N1—C6—C1	120.0 (3)	C7—C12—C11	117.4 (3)
N1—C6—C5	121.8 (3)	C12—N1—C6	118.4 (2)
C1—C6—C5	118.2 (3)	C11—N2—C5	122.4 (2)
C8—C7—C12	120.4 (3)	C11—N2—H2B	118.8
C8—C7—H7A	119.8	C5—N2—H2B	118.8
			
C6—C1—C2—C3	0.4 (5)	C9—C10—C11—C12	1.1 (5)
C1—C2—C3—C4	−1.4 (5)	C8—C7—C12—N1	−178.4 (3)
C2—C3—C4—C5	0.5 (5)	C8—C7—C12—C11	1.9 (5)
C3—C4—C5—N2	−179.0 (3)	N2—C11—C12—N1	−1.7 (4)
C3—C4—C5—C6	1.4 (5)	C10—C11—C12—N1	178.0 (3)
C2—C1—C6—N1	−178.1 (3)	N2—C11—C12—C7	178.0 (3)
C2—C1—C6—C5	1.4 (5)	C10—C11—C12—C7	−2.3 (4)
N2—C5—C6—N1	−2.4 (4)	C7—C12—N1—C6	−177.8 (3)
C4—C5—C6—N1	177.2 (3)	C11—C12—N1—C6	1.9 (4)
N2—C5—C6—C1	178.1 (3)	C1—C6—N1—C12	179.7 (3)
C4—C5—C6—C1	−2.3 (4)	C5—C6—N1—C12	0.1 (4)
C12—C7—C8—C9	−0.4 (6)	C10—C11—N2—C5	179.5 (3)
C7—C8—C9—C10	−0.9 (6)	C12—C11—N2—C5	−0.7 (4)
C8—C9—C10—C11	0.5 (6)	C4—C5—N2—C11	−177.0 (3)
C9—C10—C11—N2	−179.2 (3)	C6—C5—N2—C11	2.7 (4)

### Hydrogen-bond geometry (Å, º)

**Table:**

*D*—H···*A*	*D*—H	H···*A*	*D*···*A*	*D*—H···*A*
N2—H2*B*···Br1	0.86	2.31	3.155 (4)	167
C3—H3*A*···Br1^i^	0.93	2.82	3.750 (5)	176

Symmetry code: (i) −*x*−1, −*y*, −*z*.
